# ANKK1 is found in myogenic precursors and muscle fibers subtypes with glycolytic metabolism

**DOI:** 10.1371/journal.pone.0197254

**Published:** 2018-05-14

**Authors:** Estrella Rubio-Solsona, Salvador Martí, Juan J. Vílchez, Francesc Palau, Janet Hoenicka

**Affiliations:** 1 CIBERER Biobank, Centro de Investigación Biomédica en Red de Enfermedades Raras (CIBERER), Valencia, Spain; 2 Centro de Investigación Príncipe Felipe, Valencia, Spain; 3 Department of Neurology, Hospital Universitari i Politècnic La Fe, Valencia, Spain; 4 Neuromuscular Research Unit, Instituto de Investigación Sanitaria la Fe (IIS La Fe), Valencia, Spain; 5 Department of Medicine, University of Valencia School of Medicine, Valencia, Spain; 6 Department of Genetic and Molecular Medicine, Hospital Sant Joan de Déu, Barcelona, Spain; 7 Laboratory of Neurogenetics and Molecular Medicine, Institut de Recerca Sant Joan de Déu, Barcelona, Spain; 8 Division of Pediatrics, University of Barcelona School of Medicine, Barcelona, Spain; 9 CIBER de Salud Mental (CIBERSAM), Madrid, Spain; University of Tennessee Health Science Center College of Graduate Health Sciences, UNITED STATES

## Abstract

Ankyrin repeat and kinase domain containing 1 (*ANKK1*) gene has been widely related to neuropsychiatry disorders. The localization of ANKK1 in neural progenitors and its correlation with the cell cycle has suggested its participation in development. However, ANKK1 functions still need to be identified. Here, we have further characterized the ANKK1 localization *in vivo* and *in vitro*, by using immunolabeling, quantitative real-time PCR and Western blot in the myogenic lineage. Histologic investigations in mice and humans revealed that ANKK1 is expressed in precursors of embryonic and adult muscles. In mice embryos, ANKK1 was found in migrating myotubes where it shows a polarized cytoplasmic distribution, while proliferative myoblasts and satellite cells show different isoforms in their nuclei and cytoplasm. *In vitro* studies of ANKK1 protein isoforms along the myogenic progression showed the decline of nuclear ANKK1-kinase until its total exclusion in myotubes. In adult mice, ANKK1 was expressed exclusively in the Fast-Twitch muscles fibers subtype. The induction of glycolytic metabolism in C2C12 cells with high glucose concentration or treatment with berberine caused a significant increase in the *ANKK1* mRNA. Similarly, C2C12 cells under hypoxic conditions caused the increase of nuclear ANKK1. These results altogether show a relationship between *ANKK1* gene regulation and the metabolism of muscles during development and in adulthood. Finally, we found ANKK1 expression in regenerative fibers of muscles from dystrophic patients. Future studies in ANKK1 biology and the pathological response of muscles will reveal whether this protein is a novel muscle disease biomarker.

## Introduction

Ankyrin repeat and kinase domain containing 1 (ANKK1) protein belongs to the receptor-interacting protein (RIP) kinase family [1]. RIP kinases are involved in cell survival and death [1–2], as well as in differentiation processes in a broad variety of tissues [2–4]. ANKK1 has been related to dopaminergic transmission [5], neurogenesis [6] and psychiatric disorders [5]. In previous studies we found this protein is localized in radial glial cells [7] and other neurogenic progenitors in the brain where it is differentially expressed during the cell cycle [8]. The gene that codes for ANKK1 is located in a 512 Kb cluster along with neural cell adhesion molecule 1 (*NCAM1*), dopamine receptor D2 (*DRD2*) and tetratricopeptide repeat domain 12 gene (*TTC12*) genes [9]. The NTAD gene cluster (*NCAM1-TTC12-ANKK1-DRD2*) shows a conserved synteny and neighborhood thus suggesting the coordinated functioning of these proteins by co-regulation of gene expression [9].

RIP4, the most similar RIP in the overall structural organization to ANKK1, is also involved in developmental processes that regulate epidermal morphogenesis and cutaneous wound repair [10]. RIP2 is a checkpoint in skeletal myogenesis [11] and has to be downregulated in mouse myoblasts for correct differentiation [12]. In addition, the study of other RIP proteins during myogenesis has shown changes in expression throughout the process of myotube differentiation [12].

Immunolabeling studies of ANKK1 in the central nervous system of mouse samples have shown us that it is also expressed in embryonic myotubes [13]. Based on these observations, here we hypothesize that ANKK1 participates in embryonic myogenesis and adult muscle regeneration. Our findings in mice embryos, myogenic cell lines and adult muscles show that ANKK1 is located in specific myogenic precursors and in muscle fiber subtypes with glycolytic metabolism.

## Materials and methods

### Animals and sample preparation

BALB/cAnNHsd mice embryos (Harlan Interfauna Ibérica) or C57BL/6 mice (Jackson Laboratory Repository) at 8 weeks of age were used as tissue source. The experimental procedures were performed following the European Union Council guidelines (2010/63/EU) and Spanish regulations (RD 1201/2005) and were performed according to the Ethical Committee of *Centro de Investigación Príncipe Felipe* (Valencia, Spain). Mice were maintained at 21 ± 2°C in 12 h light/dark cycles with food and water *ad libitum*. For adult muscle studies, C57BL/6 mice were sacrificed by cervical dislocation and gastrocnemius and flexor digitorum brevis (FDB) muscles were removed and prepared as previously described with some modifications [14–15]. Gastrocnemiu*s* were fixed in 4% paraformaldehyde (PFA) in phosphate-buffered saline (PBS) for 24 h at 4°C and embedded in paraffin blocks. Transversal tissue serial sections (5 μm) were cut on a microtome (MICROM HM340E) and mounted on poly-L-lysine coated glass slides. Single myofibers of FDB muscles were obtained after 2.5 h of enzymatic digestion at 37°C with 0.2% collagenase type IV (MP Biomedicals) in Dulbecco’s Modified Eagle’s Medium (DMEM)-high glucose (Gibco) followed by gentle trituration using Pasteur pipettes rinsed with 10% horse serum (HS) (Sigma-Aldrich). To purify the myofibers from free mononucleated cells, debris and damaged myofibers, the suspension was transferred to the top of a tube containing 10 ml of DMEM-high glucose supplemented with 1% penicillin/streptomycin (Sigma-Aldrich) and 10% HS, and myofibers were allowed to settle at 1 x g during 15 min at room temperature [14]. After the third purification process, the supernatant was aspirated and myofibers cultured for 24 h in 1 mg/ml matrigel (BD Biosciences) coated dishes with DMEM-high glucose supplemented with 1% penicillin/streptomycin, 1% HS and 20% fetal bovine serum (FBS) (Sigma-Aldrich).

### Human samples

Samples of deltoid and quadriceps muscle tissue were obtained in the Neuromuscular Research Unit of the *Instituto de Investigación Sanitaria La Fe* (Valencia, Spain), collected and processed according their protocols for human studies, and approved by the Ethical Committee of *Hospital Universitari i Politècnic La Fe* (Valencia, Spain), in accordance with the Declaration of Helsinki (2013). A parent or guardian of the participants provided written informed consent and each subject provided written assent for the collection of samples and subsequent analyses. The muscles samples studied were from two non-dystrophic patients without a history of muscular disease (1 male and 1 female, 1 and 29 years of age respectively) and four dystrophic patients (1 male and 3 females; 8, 26, 30 and 61 years of age respectively). Muscle biopsies were immersed 30 seconds in cold isopentane and stored in liquid nitrogen until use. Transversal tissue serial sections (7 μm) were cut in a microtome, mounted on glass slides and stored at -20°C.

### Cell lines and treatments

C2C12 (CRL-1772, ATCC) and Rhabdomyosarcoma cells (RD, CRL-136, ATCC) were cultured in DMEM (Gibco). C2C12 was supplemented with 10% FBS, 2mM L-glutamine (Sigma-Aldrich), 1% penicillin/streptomycin while RD was supplemented with 2% MEM vitamin solution and 2% MEM non-essential amino acids (Sigma-Aldrich). Cells were maintained in a 5% CO_2_ atmosphere at 37°C. The culture medium was changed every 3 days.

To induce myoblast differentiation, C2C12 cells were changed to differentiation medium containing DMEM 2% HS [16] when they reached 80% confluence. In the case of RD, cells were changed to differentiation medium plus 2 μM insulin (Sigma-Aldrich) [17] and, after 24 h 5 μM of retinoic acid (Sigma-Aldrich) was added. The cells were harvested at different time points until 6 days of differentiation.

For the induction of glycolytic metabolism in C2C12, the cells were cultured in DMEM with glucose at high concentration (30 mM, HG) or treated with 30 μmol/L berberine (Sigma-Aldrich) as previously described [18–19]. Additionally, the induction of glycolytic metabolism was performed by decreasing oxygen levels up to 2% during 24 and 48 h.

When necessary, C2C12 cells were induced to differentiate and treated with 2 ng/ml leptomycin B (LMB, Sigma-Aldrich) for 24 h before fixed or harvested [20].

### Lactate assay and oxygen consumption

The induction of glycolytic metabolism was determined by measuring the generation of L-lactic acid in the culture medium following the instructions of the manufacturer of the L-lactic acid test kit (K-LATE, Biocon). The amount of light intensity absorbed was detected using the Victor 1420 Multilabel Hts counter spectrophotometer (Perkin Elmer) at 340 nm. Oxygen consumption was also measured using a Clark type oxygen electrode (Rank Brothers) at 37°C with a magnetic stirrer as described elsewhere [21]. The cells were trypsinized, counted and suspended in 1 ml of DMEM. Oxygen consumption is given in nmol O_2_ min^−1^ 10^6^ cells^−1^.

### RNA isolation and quantitative RT-PCR analysis

Total RNA was isolated with Trizol reagent (Invitrogen) according the instructions of the manufacturer. Maxima first strand cDNA Synthesis kit for RT-qPCR (Thermo Scientific) was used for the reverse transcriptase (RT) reaction. All quantitative real time RT-PCR reactions were carried out on a 7500 Fast Real-Time PCR System (Applied Biosystems) using FastStart Universal SYBR Green Master Rox (Roche). *Ankk1* gene expression was quantified by the standard-curve method as previously described [6, 22]. Samples were normalized to tubulin alpha 1a (*Tuba1a*) and casein kinase 2 alpha 2 (*Csnk2a2*) housekeeping genes in the same cDNA sample. The primers used to test mice samples were the following: *Ankk1* sense 5’- TCCGATTTTGGCCTGTCCAAG- 3’, *Ankk1* antisense 5’- AGATGACAATTGCAAAGCTGTAC -3’ (from nucleotide 549 to 692 in *Ankk1* mRNA gi:146198787), *Tuba1a* sense, 5’- GAACCCACGGTCATCGATGAAG -3’, *Tuba1a* antisense, 5’- CTGTGCACTGGTCAGCCAGC -3’ (from nucleotide 258 to 419 in α-*Tubulin* mRNA gi:133892260) and *Csnk2a2* sense, 5’- GTAAAAATTCTCAAGCCAGTGAAG -3’, *Csnk2a2* antisense, 5’- GCTGGTGTCTTTGACACAGGG -3’ (from nucleotide 574 to 684 in *Csnk2a2* gi:164519099).

### Immunofluorescence and immunohistochemistry

For immunofluorescence (IF) studies, cells and FDB fibers seeded on glass coverslips and slide-mounted embryos as previously described, with some modifications [6, 8, 23]. All samples were post-fixed in 4% PFA. Cells and FDB fibers were permeabilized with 100% methanol. Samples were treated with blocking solution (10% normal goat serum, 0.1% Triton X-100, 0.5% bovine serum albumin (BSA) (Sigma-Aldrich) in PBS for embryos, 5% normal goat serum, 1% BSA in PBS for cells and FDB fibers) and then probed with primary antibodies (embryo slices for 2 nights, FDB fibers overnight and cells for 1 h). ANKK1 proteins were detected with α-STk and α-STk2, raised against two peptides corresponding to the N-terminal of the predicted ANKK1 ORF [6], α-STk3, raised against a peptide corresponding to the C-terminal of the predicted ANKK1 ORF, whose specificity was also assessed as previously described [6]; and a commercial antibody α-ANKK1 human-specific (LSBio, Inc.). From each ANKK1 antibody, a dilution of 1:200 was used in immunostaining assays. The following mouse monoclonal antibodies were used: α-beta III Tubulin as a neuronal marker (1:7000, Tubb3, Promega), α–embryonic Myosin heavy chain (eMyHC) as embryonic myoblasts marker (1:80, clone F1.652-s, Developmental Studies Hybridoma Bank, DSHB); α-alpha-Actinin as myotubes marker (1:200, A7732, Sigma-Aldrich); α-Pax7 as SCs marker (1:50, Pax7-c, DSHB); α-MyoD as committed myoblasts marker (1:200, sc-32758, Santa Cruz Biotechnology). Primary antibodies were detected using goat α-mouse or goat α-rabbit antibodies coupled to Alexa Fluor 488 or Texas Red 546 for 1 h (1:1000, Jackson ImmunoResearch). All samples were mounted with DAPI Fluoromount-G (SouthernBiotech) to visualize nuclei. Cover slips were then fixed on microscope slides.

For immunohistochemistry (IHC) studies, the murine muscle sections were pretreated to dewaxing and rehydration. The human and murine sections were then fixed with 4% PFA and permeabilized with 100% methanol/H_2_O_2_ (29:1). For antigen retrieval tissue sections were boiled for 10 min in citrate buffer (100 mmol/L sodium citrate solution, 100 mmol/L citric acid solution, pH 6.0, Sigma-Aldrich) with the exception of ANKK1 antibodies. In the former, tissue sections were incubated with 0.2% collagenase IV in DMEM for 40 min at 37°C. All sections were blocked (10% FBS, 3% BSA in PBS) and then incubated with primary antibodies overnight: a mix of α–embryonic and neonatal Myosin was used as regenerating fibers markers (eMyHC: 1:20; MyHCn: 1:20, NCL-MHCn, Leica Byosistems); α-MyHC1 and α-MyHC2 as type 1 and 2 muscle fibers markers, respectively (MyHC1: 1:200, NOQ7.5.4D, Invitrogen; MyHC2: 1:200, M4276, Sigma-Aldrich). Primary antibodies were detected using goat α-mouse or goat α-rabbit antibodies coupled to biotin for 45 min (1:1000, Jackson ImmunoResearch). DAB Peroxidase Substrate Kit (Vector) was used for antigen detection according to manufacturer’s protocol and sections were stained with hematoxylin.

### Western blot analysis

Western blot has been performed following previous protocols [6]. Briefly, to obtain membrane and cytoplasmic protein-enriched fractions, cells were homogenized in lysis buffer (150 mM NaCl, 20 mM Tris HCl, 5 mM EDTA, 10% glycerol, 1% NP-40) containing a protease inhibitor cocktail (Complete Mini-Protease Inhibitor Cocktail, Roche) and centrifuged for 100.000 x g, 4°C. The final supernatant was used as the cytoplasmic fraction and the pellet was resuspended in lysis buffer containing 0.5% sodium dodecyl sulphate (SDS) and used as the nuclear fraction. Genomic DNA was sheared by sonication with a Microson Ultrasonic Cell Disruptor (Misonix). Equal amounts of protein samples (40 μg) quantified by Pierce BCA Protein Assay Kit (Thermo Scientific) were resolved on 9% SDS-PAGE and transferred to nitrocellulose membranes (Whatman). After overnight blocking (5% nonfat dry milk and 0.1% Tween 20 in Tris buffered saline (TBS)), the membranes were incubated with the primary antibody for 1 h: α-STk2 (1:2000) and α-STk3 (1:500) to detect ANKK1, α-beta-Actin as a reference to quantify the relative protein expression level (1:5000, A1978, Sigma-Aldrich), α-alpha-Tubulin (1:10000, T9026, Sigma-Aldrich) as a cytosolic marker, α-Lamin as a nuclear marker (1:4000, sc-7292, Santa Cruz Biotechnology) and α-MyoD (1:2000). All washing steps were performed with TBS-0.1% Tween 20. Primary antibodies were detected using goat α-mouse or goat α-rabbit antibodies coupled to peroxidase for 1 h (1:10000, Thermo Scientific). Proteins were processed for chemiluminescence with the Clarity Western ECL Substrate (Bio Rad) and scanned. Bands intensity was measured through the total area of the peaks using the ImageJ 1.45v software from National Institutes of Health.

### Image acquisition

Sections from IHC were visualized with a DMR microscope while fluorescent images were taken using a SP8 scanning laser confocal microscope (Leica Microsystems). Sections of negative control tissue incubated with secondary antibodies were used for background setting previous to image acquisition. All images were captured under constant exposure time, gain, and offset. One hundred cells from 3 independent experiments were counted for each condition to calculate an average of the fluorescence observed patterns. The images were analyzed and processed (maximum intensity projections of Z-series) using ImageJ 1.45v.

### Statistics

All statistics were performed using GraphPad Prism v5.01. Normal distribution of data was tested using the Kolmogorov-Smirnov test. Cell treatment experiments were analyzed using Student’s *t* test and degrees of freedom were determined. Data are expressed as the mean ± standard deviation and *p* values less than 0.05 were considered significant.

## Results

### ANKK1 is involved in myogenic differentiation

**Fig 1:**
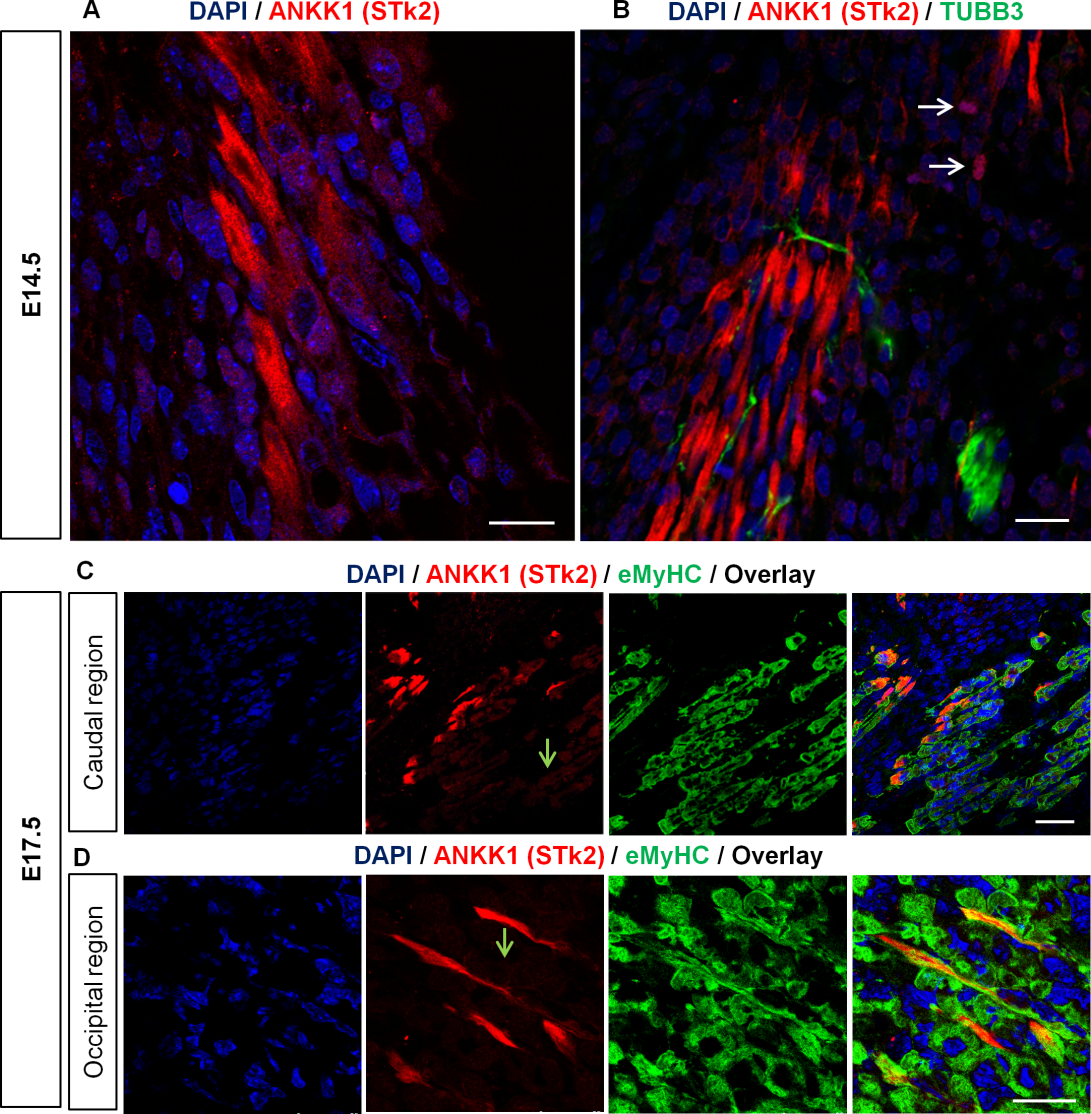
ANKK1 is located in migrating myoblasts and myotubes during development. ANKK1 (α-STk2), TUBB3 and eMyHC immunostaining of sagittal sections of mice embryos at E14.5 (A-B) and E17.5 (C-D). **(A)** ANKK1 is expressed in protrusions of embryonic myotubes. Scale bar: 25 μm. **(B)** ANKK1 staining of myotubes clusters and TUBB3+ neural precursors. The white arrows indicate ANKK1+ nuclei. Scale bar: 25 μm. **(C)** Colocalization of ANKK1 and eMyHC in myotubes derived from the dorsal portion of caudal and **(D)** occipital muscle anlagen. The green arrows show ANKK1− myotubes. Scale bar: 50 μm. Images were taken from confocal optical sections that are representative for the group averages.

To study *ANKK1* gene expression pattern during muscles differentiation we performed immunolabeling experiments using α-STk2 antibody (recognizes ANKK1 kinase domain) [6], and α-eMyHC in mice embryos at E14.5 and E17.5. A high level of ANKK1 signal was detected in embryonic myotubes distributed throughout the neuraxis, including the trunk and limb, as well as various cervical and facial muscles anlagen (Fig 1). The striated cardiac and the smooth muscle were negative for ANKK1 staining.

At the subcellular level, ANKK1 was located in the cytoplasm of myotubes with migratory morphology. In the caudal region, myotubes protrusions (Fig 1A and 1B) showed an intense ANKK1 signal that decreased as it entered the myotube producing a polarized pattern. Double immunolabeling with α-eMyHC revealed that ANKK1 was expressed in a subpopulation of myotubes derived from both muscle anlagen of caudal (Fig 1C) and occipital regions (Fig 1D). The formers showed the ANKK1 signal in myotubes with polarized migratory morphology (Fig 1D). None of the myotubes were ANKK1+ at their nuclei, however we observed nuclear ANKK1 in some nearby cells (Fig 1B).

**Fig 2:**
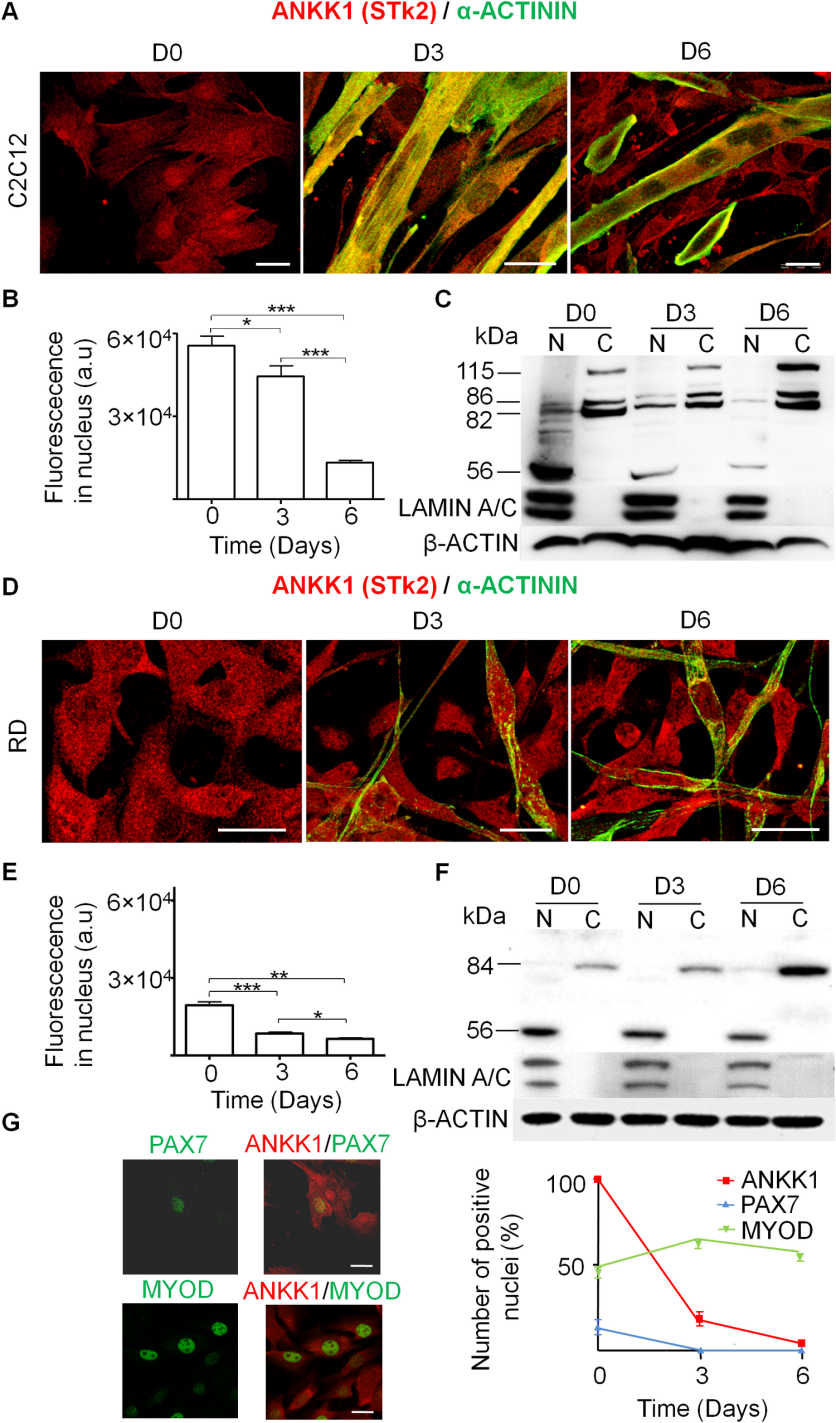
ANKK1 participates in myogenic differentiation. **(A)** ANKK1 (α-STk2) and ACTININ immunostaining of C2C12 cells during myogenic differentiation. D0: proliferative myoblast; D3: myoblasts in differentiation; D6: myotubes. **(B)** Quantification of nuclear ANKK1 fluorescence intensity (a.u.) (N = 3) **(C)** Western blot analysis of C2C12 subcellular fractions. β-ACTIN was used as control and α-LAMIN A/C as nuclear marker (N = 1). **(D)** Rhabdomyosarcoma (RD) myoblasts differentiation, **(E)** quantification of nuclear ANKK1 (N = 3) and **(F)** Western blot of subcellular fractions (N = 1). **(G)** Immunostaining of ANKK1 with PAX7 and MYOD in C2C12 cells, (left) and quantification of positive nuclei for ANKK1, PAX7 and MYOD along myogenic differentiation (right, N = 3). Images were taken from confocal optical sections that are representative for the group averages. Scale bar: 25 μm *p* < 0.05: *; *p* < 0.01: **; *p* < 0.001: ***. D: Days after induction of differentiation; a.u: arbitrary units; N: Nucleus; C: Cytoplasm.

To study the ANKK1 isoforms expression pattern during myogenic differentiation, the primary cell line of murine myoblasts C2C12 was differentiated to myotubes (Fig 2A–2C). Immunolabeling studies with α-STk2 (ANKK1) and α-Actinin as a marker of differentiating skeletal muscle cells showed different expression patterns of ANKK1 throughout the differentiation process. We observed ANKK1 signal both in the nucleus (greater intensity) and in the cytoplasm (lower intensity) of proliferating C2C12 myoblasts (Fig 2A). The nuclear signal of ANKK1 declined along the myogenic progression until its total exclusion in myotubes (Fig 2A). The quantification of ANKK1 signal at the nuclei (Fig 2B) confirmed the significant downregulation of the protein after 3 and 6 days (D) of myoblast differentiation (D0-D3, t: 2.639, *p* = 0.0147; D0-D6, t: 13.93, *p* <0.0001; D3-D6, t: 8.157, *p* <0.0001). In addition, Western blot studies of C2C12 subcellular protein fractions confirmed the exclusion of ANKK1 from the myotubes nuclei (Fig 2C). Moreover, we observed differences in subcellular location of ANKK1 isoforms. ANKK1-kinase (~56- kDa band) was mainly localized at the nucleus while ANKK1 full-length isoform (~82- kDa band) at the cytoplasm (Fig 2C). We also found additional ~86 and ~115- kDa bands at the cytoplasm fraction, the latter would correspond to a glycosylated ANKK1-full length isoform (S1 Fig). The study of ANKK1 proteins during myogenic differentiation of human rhabdomyosarcoma (RD) cell line also shows a decrease in the nuclear signal (D0-D3, t: 6.137, *p* <0.0001; D0-D6, t: 8.620, *p* <0.0001; D3-D6, t: 2.591, *p* = 0.0179) (Fig 2D–2F). In addition, immunolabeling staining of C2C12 cells with α-STk2, along with α-Pax7 and α-MyoD was performed to study a possible relationship between ANKK1 and these nuclear markers of the initial myogenic steps (Fig 2G). In proliferative conditions, ANKK1 signal with variable intensity colocalized with PAX7+ (13.82%) and MYOD+ cells (45.4%). When myoblast differentiation was induced, at D3 we observed a significant downregulation of ANKK1 (t: 46.21, *p* <0.0001) and PAX7 signal at the nucleus (18.62 and 0%, respectively) while MYOD was increased (t: 2.883, *p* = 0.0067) (62.29%). After 6 days in differentiation medium, MYOD showed a small decreased to 54.4%: D0-D6 (t: 1.557, *p* = 0.1285), D3-D6 (t: 2.283, *p* = 0.0365) while ANKK1 was reduced to 3.94% of the cells: D0-D6 (t: 313.5, *p* <0.0001), D3-D6 (t: 2.462, *p* = 0.0433). Therefore, the study of ANKK1 isoforms in cell-derived myoblasts/myotubes showed a phase-specific expression pattern during myogenic progression.

### Leptomycin B inhibits the nuclear export of ANKK1 during myoblast differentiation

**Fig 3:**
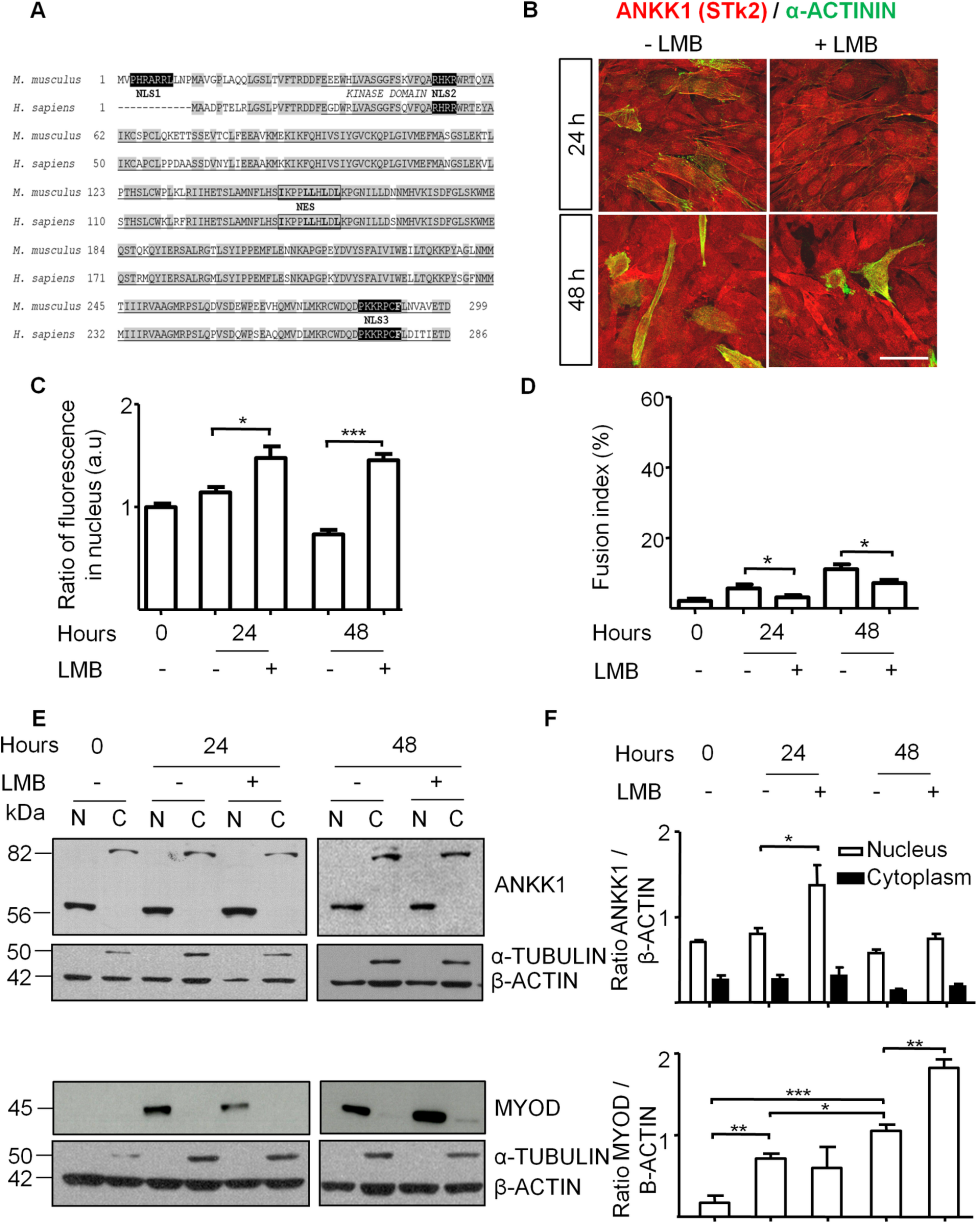
Leptomycin B inhibits the nuclear export of ANKK1 during myoblast differentiation. **(A)** Alignment analysis of predicted amino acid sequences of ANKK1 kinase domain in *Mus Musculus* (GenBank: AAI45079.1) and *Homo sapiens* (NP_848605.1) and identification of NLS and NES. Shading indicates 100% sequence identity. The kinase domain is underlined. Highly hydrophobic amino acids in NESs are marked in bold. **(B)** ANKK1 (α-STk2) and α-ACTININ immunostaining of C2C12 myoblasts in differentiation (24–48 h) treated with 2 ng/ml LMB 24 h before fixed. Scale bar: 25 μm. Images were taken from confocal optical sections that are representative for the group averages. **(C)** Quantification of ANKK1 fluorescence intensity (a.u) in nucleus (N = 3) and **(D)** fusion index (N = 3). **(E)** Western blot of subcellular C2C12 fractions using α–STk2 and α–MyoD. β-ACTIN was used as control and α-TUBULIN as cytosolic marker. **(F)** Western blot bands were quantified using the ImageJ 1.45v software and the relative expression was represented related to β-ACTIN value. *p* < 0.05: *; *p* < 0.01: **; *p* < 0.001: ***. a.u: arbitrary units; N: Nucleus; C: Cytoplasm.

We have previously reported *in silico* evidence for nuclear localization signals (NLS) and nuclear export sequences (NES) on human ANKK1 [24]. Here the *in silico* analysis of mice ANKK1 using the NUCDISC program (from the PSORT II server, http://psort.ims.u-tokyo.ac.jp) and NEtNES v1.1 server revealed unique and homologous consensus NLS and NES sequences when compared with the human protein (Fig 3A). The unique NLS in the mouse ANKK1 comprised amino acids 3 to 9 (PHRARRL). These *in silico* results suggest that nuclear exclusion of ANKK1 in myotubes could be mediated by NES. Then, we studied the effect of LMB, an inhibitor of nuclear export of NES containing proteins, on the subcellular localization of ANKK1 during differentiation.

C2C12 myoblasts were induced to undergo myotubes formation during 24 or 48 h and treated with LMB 24 h before fixed or harvested. After treatment, we observed a significant increase of ANKK1 signal in myoblasts nuclei and inhibition of the fusion of myotubes when compared to controls (Fig 3B). Specifically, C2C12 nuclei of treated myoblasts showed higher ANKK1 levels at both 24 (t: 2.728, *p* = 0.0117) and 48 h (t: 10.37, *p* <0.0001) (Fig 3C), and a significant reduction in the fusion index (ratio between number of Actinin+ cells/total cells) at 24 (t: 2.135, *p* = 0.0441) and 48 h (t: 2.564, *p* = 0.0150) (Fig 3D). We also performed, in LMB-treated and untreated C2C12 cells, Western blot analysis of subcellular fractions using α–STk2 and α–MyoD (Fig 3E and 3F), and observed a significant nuclear ANKK1-kinase isoform increase after LMB treatment (Fig 3E) at 24 h (t: 2.505, *p* = 0.0336) (Fig 3F) while cytoplasmatic ANKK1 full-length isoform levels remained unchanged in all conditions: 24 h (t: 0.3746, *p* = 0.7158); 48 h (t: 1.069, *p* = 0.3455). This result revealed that nuclear ANKK1-kinase retention by LMB in early differentiating myoblasts did not affect the full length isoform expression. In addition, ANKK1 showed more sensitivity to LMB treatment in comparison to MYOD (Fig 3E), although a significant increase of the former was evident after 48 h (t: 5.901, *p* = 0.0041) (Fig 3F). These findings demonstrate that LMB inhibits the nuclear export of ANKK1. In addition, ANKK1 expression pattern shows an inverse relationship to MYOD expression in early stages of differentiation.

### ANKK1 is a marker of SCs and fiber subtype in adult muscles

**Fig 4:**
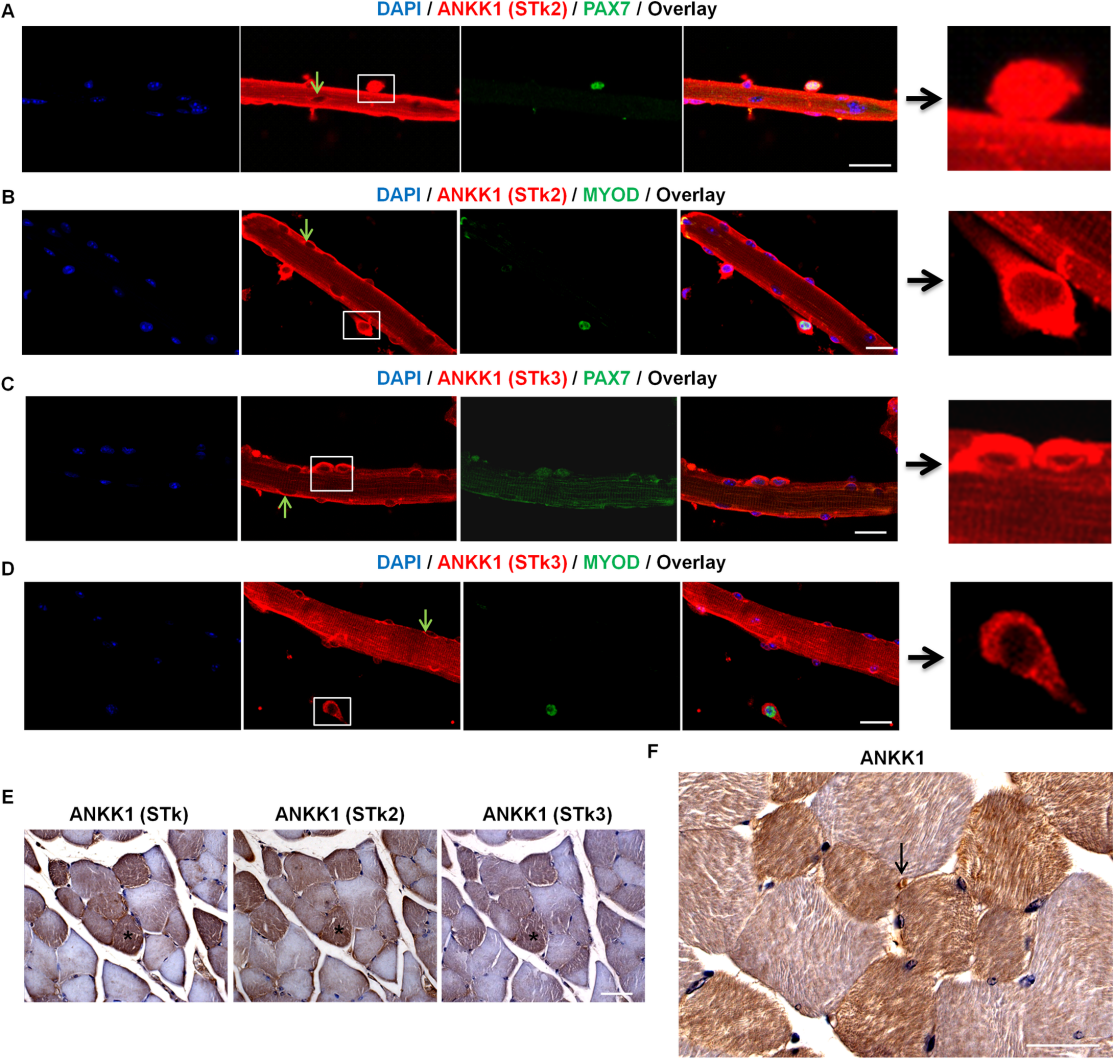
ANKK1 is located in SCs and in a fiber subtype in adult muscles. ANKK1 (detected with α-STk, α-STk2 and α-STk3), PAX7 and MYOD immunostaining of mouse FDB isolated fibers and gastrocnemius sections of adult mice. Nuclei were stained with DAPI in IF and with hematoxylin in IHC. **(A)** ANKK1+ (α-STk2)/PAX7+ SCs. Boxed area in ANKK1 images are amplified [25]. The green arrows show ANKK1− nuclei. **(B)** ANKK1+(α-STk2)/MYOD+ SCs showing ANKK1 cytoplasmic expression. **(C)** ANKK1+(α-STk3)/PAX7+ and **(D)** ANKK1+(α-STk3)/MYOD+ SCs showing ANKK1 cytoplasmic expression. Scale bar: 25 μm. **(E)** α-STk, α-STk2 and α-STk3 antibodies recognize the same pattern for ANKK1 in a serial of transversal gastrocnemius sections. **(F)** ANKK1 is expressed in the cytoplasm of some fibers and in SCs (black arrow). Scale bar: 50 μm. Images are representative for the group averages.

ANKK1 expression pattern studies in muscles of adult mice were performed using FDB isolated fibers and transversal gastrocnemius serial sections. In FDB fibers, ANKK1 is located at cytoplasm and perinuclear regions, although it is totally absent in myonuclei (Fig 4). Immunolabeling experiments with α–STk2, α–Pax7 and α–MyoD showed ANKK1 expression in the nucleus and cytoplasm of all PAX7+ SCs (Fig 4A) whereas the location of the protein was exclusively cytoplasmic in MYOD+ SCs (Fig 4B). To know whether the ANKK1-full length isoform is expressed in the cytoplasm of SCs, we used the α–STk3 antibody (Fig 4C and 4D) that recognizes the Ankyrin repeats domain of ANKK1 which is absent in ANKK1-kinase isoform. We found ANKK1 signal in the cytoplasm of PAX7+ and MYOD+ SCs (Fig 4C and 4D). Therefore, the location of ANKK1 isoforms in SCs was identical to that observed in myoblasts and cell-derived myotubes. In the same way, the study of ANKK1 in mice gastrocnemius sections using α–STk, α-STk2 and α–STk3 [6] (Fig 4E) showed an identical expression pattern of ANKK1. Specifically, ANKK1 was expressed in the cytoplasm of a subpopulation of muscle fibers (Fig 4F). Further, fibers myonuclei were ANKK1−, while we observed some ANKK1+ positive nuclei (Fig 4F), that would correspond to the SCs. Therefore, in adult mice ANKK1 is expressed in SCs and a subtype of muscle fibers.

### ANKK1 is exclusively expressed in Fast-Twitch muscle fibers

**Fig 5:**
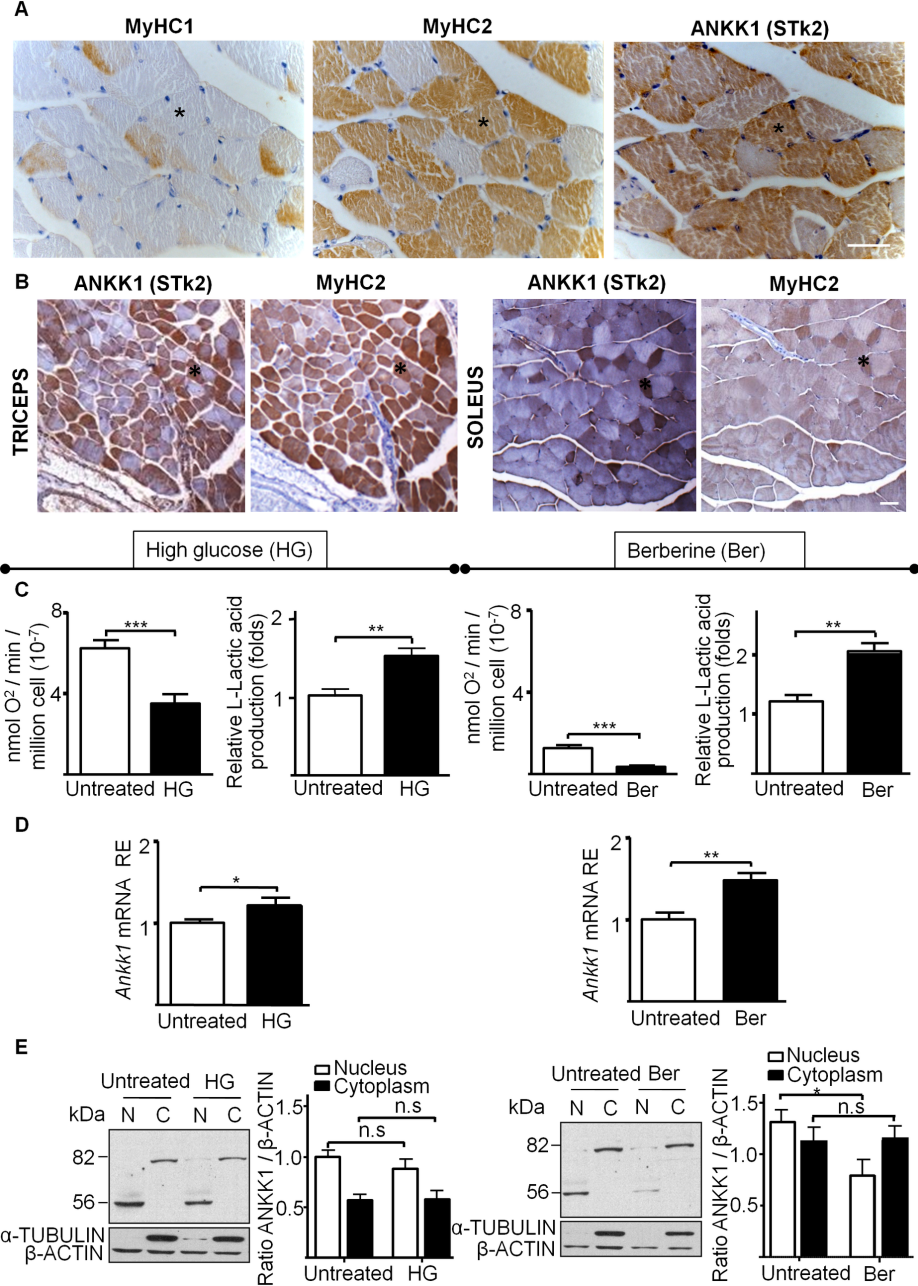
*ANKK1* is expressed exclusively in Fast-Twitch fibers. **(A)** Representative images for the group averages of IHC analysis of ANKK1, MyHC1 and MyHC2 in mice adult skeletal gastrocnemius serial sections and **(B)** colocalization of ANKK1 and MyHC2 in triceps and soleus muscles. The black asterisk indicates the same fiber. Scale bar: 50 μm. **(C)** Glycolysis induction in C2C12 myoblasts using HG and berberine during 24 h treatments. Oxygen consumption was assessed using a Clark-type oxygen electrode (N = 7) and lactic acid production was measured (N = 3). Relative oxygen and L-lactic acid production values are shown (±SD). **(D)** *ANKK1* expression analysis by reverse transcriptase polymerase chain reaction (N = 3). Relative mRNA values (±SD) are shown. **(E)** Western blot of subcellular C1C12 fractions using α-STk2 (N = 4). β-ACTIN was used as control and α-TUBULIN as cytosolic marker. Western blot bands were quantified using the ImageJ 1.45v software and the relative expression was represented related to β-Actin value. *p* < 0.05: *; *p* < 0.01: **; *p* < 0.001: ***.N: nucleus; C: cytoplasm; RE: Relative Expression; Ber: berberine.

To determine if the pattern of the differential expression of ANKK1 in adult muscle was related to fiber type, serial sections of mice transversal gastrocnemius muscles were studied using IHC staining for detection of ANKK1 (α-STk2), MyHC1 and MyHC2 (Fig 5A). We found ANKK1 signal in most of the MyHC2+ fibers (Fig 5A) while the protein was absent in the MyHC1+ oxidative fibers. ANKK1 was also found in MyHC2+ fibers in triceps (more glycolytic muscle [26]) and soleus (more oxidative muscle [27]) (Fig 5B).

To know the influence of the metabolic environment on the selective ANKK1 expression in the muscles, we induced glycolytic metabolism in C2C12 by growing the cells with HG (30 mM glucose) or treating them with berberine (30 μM). A significant reduction in cellular oxygen consumption and an increase in lactic acid production were found in both conditions (Fig 5C). *ANKK1* mRNA levels showed a significant increase with HG (t: 2.289, *p* = 0.0428) and berberine treatment (t: 3.834, *p* = 0.0021) (Fig 5D). However, Western blot of C2C12 subcellular fractions showed no significant changes for ANKK1-kinase (t: 0.9711, *p* = 0.3599) or full-length isoform (t: 0.0075, *p* = 0.9418) after culture in HG (Fig 5E). By contrast, in the case of berberine we observed a significant decrease of ANKK1-kinase for the nuclear enriched fraction (t: 2.626, *p* = 0.0392) while no changes were found for the full-length isoform (t: 0.1405, *p* = 0.8918) (Fig 5E). We also attempted to induce glycolytic metabolism in C2C12 cells by cultivating them under hypoxic conditions (2% oxygen) (S2 Fig). Immunolabeling experiments with α-STk2 show a significant increase of ANKK1 in the nucleus of hypoxic cells after 24 (t: 5.802, *p* <0.0001) and 48 h (t: 6.995, *p* <0.0001, S2 Fig). Taking altogether these results, we propose that there is a relation between *ANKK1* gene expression and glycolytic metabolism that explains the specific location of the protein in Fast-Twitch muscle fibers.

### ANKK1 is expressed in regenerating fibers in muscular dystrophies

**Fig 6:**
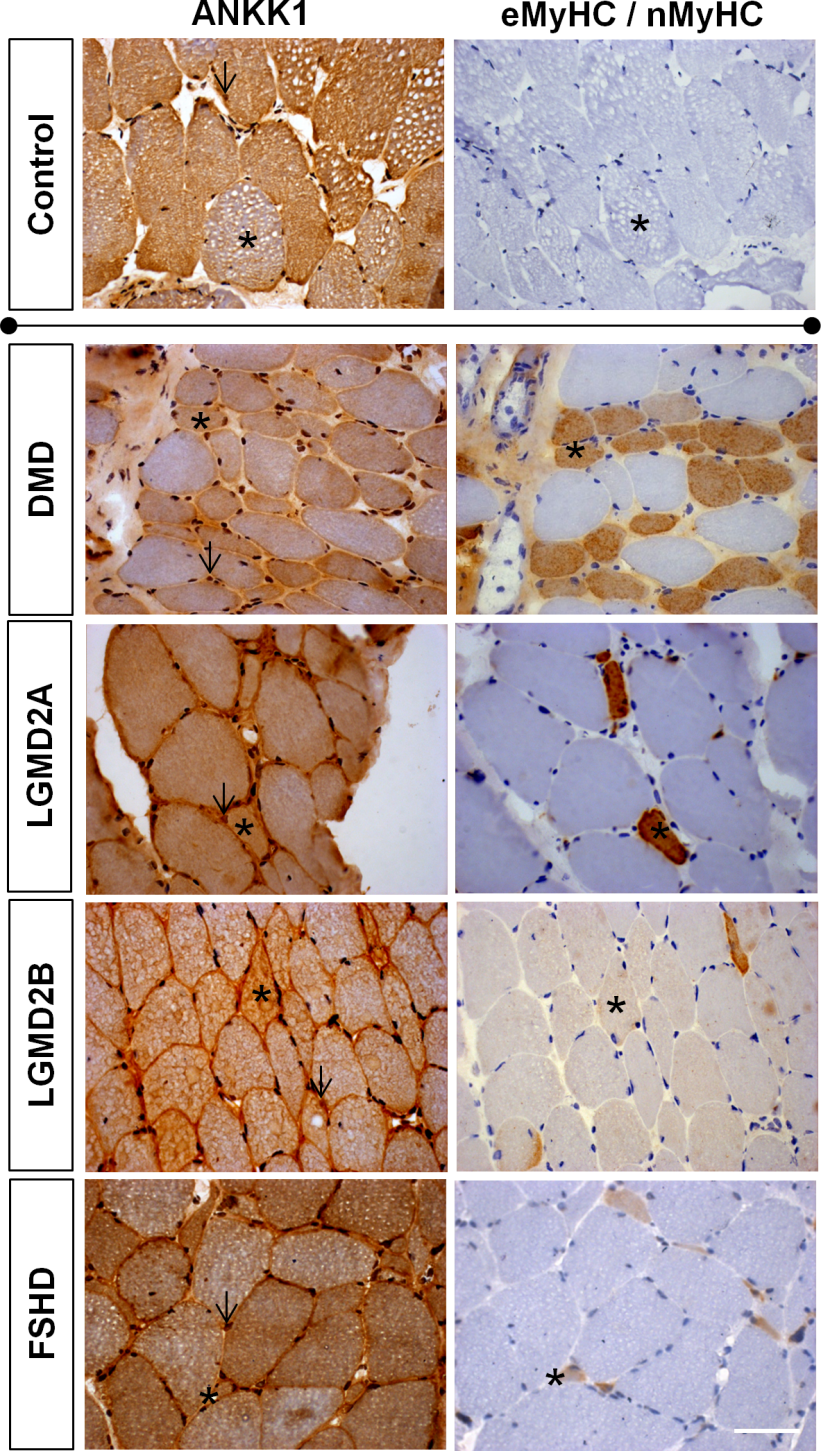
ANKK1 is expressed in regenerating fibers in neuromuscular dystrophies. ANKK1 and a mix of embryonic and neonatal myosin heavy chains (eMyHC/nMyHC) immunostaining of adult skeletal muscle sections. The samples were non-dystrophic (control) and dystrophic patients (DMD, LGDM2A, LGMD2B and FSHD). ANKK1 and eMyHC/nMyHC were studied in serial muscle sections. The black asterisk indicates the same fiber. The black arrows indicate ANKK1+ nuclei. Scale bar: 50 μm. All regenerating fibers from each tissue section were counted and analyzed. Images are representative for the group averages.

Given that ANKK1 is found in muscle precursors during development and adulthood, we aimed to investigate the expression of ANKK1 in clinical samples of muscular dystrophies. We performed IHC detection of ANKK1 using a commercial antibody against a human epitope, since the antibodies used in previous experiments did not work in human adult muscle. Colocalization experiments were done in serial sections of human quadriceps and deltoid muscles using eMyHC/nMyHC as a marker of regenerating fibers. The samples were 2 controls and 4 patients with different muscular dystrophies. Specifically, we included Duchenne muscular dystrophy (DMD, MIM #310200), autosomal recessive limb-girdle muscular dystrophy 2A (LGMD2A, MIM #253600), limb-girdle muscular dystrophy 2B (LGMD2B, MIM #253601) and facioscapulohumeral dystrophy 1 (FSHD1, MIM #158900). Non-dystrophic muscle samples showed ANKK1 expression in a subpopulation of muscle fibers and the absence eMyHC/nMyHC expression (Fig 6). Regarding dystrophic patients, ANKK1 was expressed in 100% of regenerating fibers in DMD, LGMD2A and LGMD2B samples and 71.43% in FSHD1 muscle (Fig 6).

We also quantified the number of ANKK1+ nuclei in both non-dystrophic and dystrophic samples. Non-dystrophic control fibers showed 5.88% ANKK1+ nuclei, while the percentage increased significantly in dystrophic samples: 25.74% in DMD (t: 9.053, *p* = 0.0003), 10.9% in LGMD2A (t: 2.86, *p* = 0.0459), 22.3% in LGMD2B (t: 4.288, *p* = 0.0128) and 18.12% in FSHD (t: 5.917, *p* = 0.002). These observations showed that ANKK1 is expressed in human regenerating cells and fibers under certain pathological dystrophic muscles.

## Discussion

This study shows for the first time that *ANKK1* expression is not restricted to neural precursors and postmitotic neural cells but extends to the myogenic lineage. We found ANKK1 both *in vivo* in embryonic myotubes and in adult muscles and *in vitro* in myoblasts and fusing myotubes. Our results of the study of ANKK1 in muscles are the following: first, during the development ANKK1 is located in migrating myotubes where it shows a polarized cytoplasmic distribution; second, myoblasts and SCs, that are muscle precursors, show ANKK1 in their nuclei and cytoplasm while myotubes and muscle fibers only in their cytoplasm; third, in adulthood, ANKK1 is exclusively expressed in Fast-Twitch muscle fibers subtype; and fourth, there is a relation between glycolytic metabolism and the regulation of *ANKK1* gene.

Early studies of ANKK1 in mice embryos showed that this protein is localized in the cytoplasm of the radial glia [6], which are the cells that guide migrating neurons of the developing central nervous system [28]. In this work, we found that embryonic migrating myotubes also expressed ANKK1. Therefore it would be possible that ANKK1 participates in common molecular mechanisms of migration in both cell lineages. ANKK1 shows cytoplasmic location in cell-derived and in embryonic myotubes but not with the same pattern. In cell-derived myotubes ANKK1 localizes throughout the cytoplasm whereas migrating embryonic myotubes shows the protein in a pattern of apical polarization (Fig 1A). This polarized morphology in protrusions of the leading edge of myotubes clusters is characteristic of the front of the migrating cells [29–30]. These findings show that ANKK1 has a role in the polarization of migrating cells during development, which is the first step of the migration process [31]. It is still unknown how ANKK1 contributes to cell polarization; in any case, it is located in the front of myotubes clusters that are collectively migrating (Fig 1A–1C) or in individual migrating myotubes (Fig 1D). In addition, ANKK1 is not expressed in all embryonic myotubes, but those that show more elongated morphologies and characteristic protrusions of migration. In skeletal muscle development, migration is necessary for the correct localization of cell precursors in somites to adopt myogenic potential [32] and for the fusion of myoblasts along primary and secondary myogenesis [33]. Given that cell migration is critical for normal embryonic development and functioning of an adult organism, the expression pattern of ANKK1 in the radial glia and in the embryonic myotubes would signal a role for this protein for efficient migration.

Previous studies of ANKK1 isoforms during brain development in mice had indicated that ANKK1-kinase is mainly expressed during proliferative states, whereas an inverse pattern with a prevalence of the ANKK1 full-length isoform was found in stages of neurons maturation [8]. Here, we found an equivalent ANKK1 expression pattern in mice and human myogenic cell lines (C2C12 and rhabdomyosarcoma). During the proliferative stages ANKK1-kinase was found in myoblast nuclei, where it is downregulated throughout the differentiation process, while the myotubes show a prevalence of ANKK1 full-length isoform in their cytoplasm. Accordingly, we observed some ANKK1+ nuclei in cells nearby embryonic myotubes that would correspond to muscle precursors and the lack of expression at the nuclei of adult muscle fibers. Therefore, there seems to be a relationship between ANKK1 subcellular location and the cell cycle. Indeed, we previously reported that, during the cell cycle, ANKK1 is involved in G1 and M phases in different cell lines as well as in M phase, both in neurogenesis and in self-renewal neural progenitors from mice embryo forebrain [8]. Therefore it is plausible that ANKK1 expression in myoblasts nuclei in proliferative stages has an impact upon the cell cycle and its exit during muscle differentiation. Further, we also observed in differentiating C2C12 cells a reverse relationship between nuclear ANKK1 and the myogenic factor MYOD levels in a temporal coordination between cell cycle exit and differentiation. This reverse relationship was also evident after 24 h treatment with LMB that significantly retains nuclear ANKK1 while no effect was found for MYOD. We then propose that ANKK1 nucleus-cytoplasmic shuttle is carried out by exportins before the increase of MYOD in the progression of the myogenic lineage.

The ANKK1 study in adult muscle fibers shows that it is expressed exclusively in the cytoplasm of Fast-Twitch fibers, thus suggesting a linkage between the regulation of the *ANKK1* gene and the metabolic environment. Fast-Twitch fibers obtain energy from stored glycogen and phosphocreatine, and they rather use a glycolytic metabolism [34–35]. Here we demonstrated that the induction of a glycolytic metabolism by HG levels or hypoxic conditions in C2C12 causes an *ANKK1* upregulation that could explain its expression pattern in muscles. In the case of berberine treatment, the decrease observed in nuclear ANKK1 could be explained by other pathways apart from glycolysis that are activated. For example, berberine activates myoblasts the differentiation through MYOD and p38 MAPK [36], and an inverse relationship between ANKK1 and MYOD was found. Besides, we also found ANKK1 expression in SCs that are the adult myogenic precursors [37–38]. The comparison of ANKK1 isoforms expression pattern (nuclear ANKK1-kinase *vs* cytosolic ANKK1 full-length) between quiescent/self-renewing and already committed SCs shows striking similarities with neural [39] and muscle cell precursors during development. Hence, it is tempting to consider that the *ANKK1* expression pattern in SCs could be related to metabolic changes and/or entry or exit of these cells into the cell cycle. In any case, our results in embryonic precursors and adult muscles indicate that *ANKK1* expression in the myogenic lineage is related to glycolytic metabolism.

Many similarities have been described between embryonic and adult myogenesis [40–41]. The finding that ANKK1 is expressed in human SCs of healthy muscles and, in SCs and the regenerating muscle fibers of dystrophic patients, would place this protein in overall mechanisms for muscle generation/repair. From a genetic point of view, the *ANKK1* gene location in the NTAD genomic cluster where the *NCAM1* gene maps also supports a function of this *locus* upon myogenesis. In addition to the well-established role of NCAM1 in neurogenesis, this protein has also been identified in embryonic myoblasts [42], adult SCs [43] and regenerating fibers in different neuromuscular pathologies [44–46]. Therefore, the findings of ANKK1 and NCAM1 in embryonic and adult myogenesis extend the role of the NTAD cluster beyond neurodevelopment to other cell lineages during development. Finally, since ANKK1 belongs to the RIP family and downregulation *of RIP2* in myogenesis is a prerequisite for myoblasts differentiation [12], the need to know the part played by ANKK1 warrants further studies.

In conclusion, we are providing the first evidence of ANKK1 expression in the myogenic lineage during development and in adulthood. The characteristic expression pattern of ANKK1 in myogenic differentiation and regeneration would make this protein a useful marker of muscle fiber status.

## Supporting information

S1 Fig Study of ANKK1 full-length glycosylation. **(A)** Amino acid sequence of the mouse ANKK1 full-length isoform. Identification of predicted post-translational modification sites with NetOGlyc 4.0. Putative amino acids to be glycosilated (Serine) are underlined in red. **(B)** Western blot analysis of C2C12 myotubes extracts. ANKK1 (α–STk3) detection prior and after *in vitro* deglycosilation treatment (N = 1). β-Actin was used as control. ~115-kDa band intensity decreased after deglycosilation of C2C12 myotubes compared to control.
(PDF)

S2 Fig Nuclear ANKK1 levels increase under hypoxic conditions. **(A)** ANKK1 (α-STk2) immunostaining of C2C12 proliferating myoblasts (24–48 h) in normoxia or hypoxia (2% O_2_). Images were taken from confocal optical sections that are representative for the group averages. **(B)** Quantification of the fluorescence intensity (a.u) of ANKK1 in nuclei (N = 3). *p* < 0.05: *; *p* < 0.01: **; *p* < 0.001: ***. a.u: arbitrary units.
(PDF)
