# Correction: ANKK1 is found in myogenic precursors and muscle fibers subtypes with glycolytic metabolism

**DOI:** 10.1371/journal.pone.0198880

**Published:** 2018-06-06

**Authors:** Estrella Rubio-Solsona, Salvador Martí, Juan J. Vílchez, Francesc Palau, Janet Hoenicka

In the Funding section, the following funder is missing: FEDER. The correct funding information is as follows: This work has been supported by the Fundación Isabel Gemio (http://www.fundacionisabelgemio.com) (JJV and FP), Instituto de Salud Carlos III (ES) grant PI15/01013 (JH) and FEDER. The funders had no role in study design, data collection and analysis, decision to publish, or preparation of the manuscript.
