# Supplementary figures and images for: ANKK1 is found in myogenic precursors and muscle fibers subtypes with glycolytic metabolism
Source: PLoS One. 2018 May 14;13(5):e0197254. doi: 10.1371/journal.pone.0197254 (PMC5951577; doi:10.1371/journal.pone.0197254)

## Supplementary Figure 1

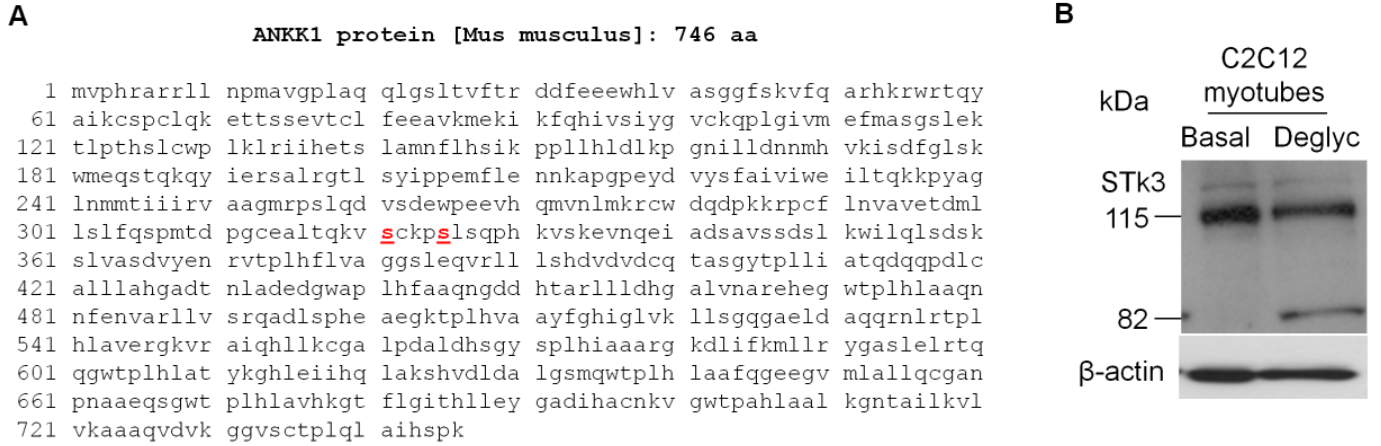

Supplement: S1 Fig — (A) Amino acid sequence of the mouse ANKK1 full-length isoform. Identification of predicted post-translational modification sites with NetOGlyc 4.0. Putative amino acids to be glycosilated (Serine) are underlined in red. (B) Western blot analysis of C2C12 myotubes extracts. ANKK1 (α–STk3) detection prior and after in vitro deglycosilation treatment (N = 1). β-Actin was used as control. ~115-kDa band intensity decreased after deglycosilation of C2C12 myotubes compared to control. (PDF) [file pone.0197254.s001.pdf]

## Supplementary Figure 2

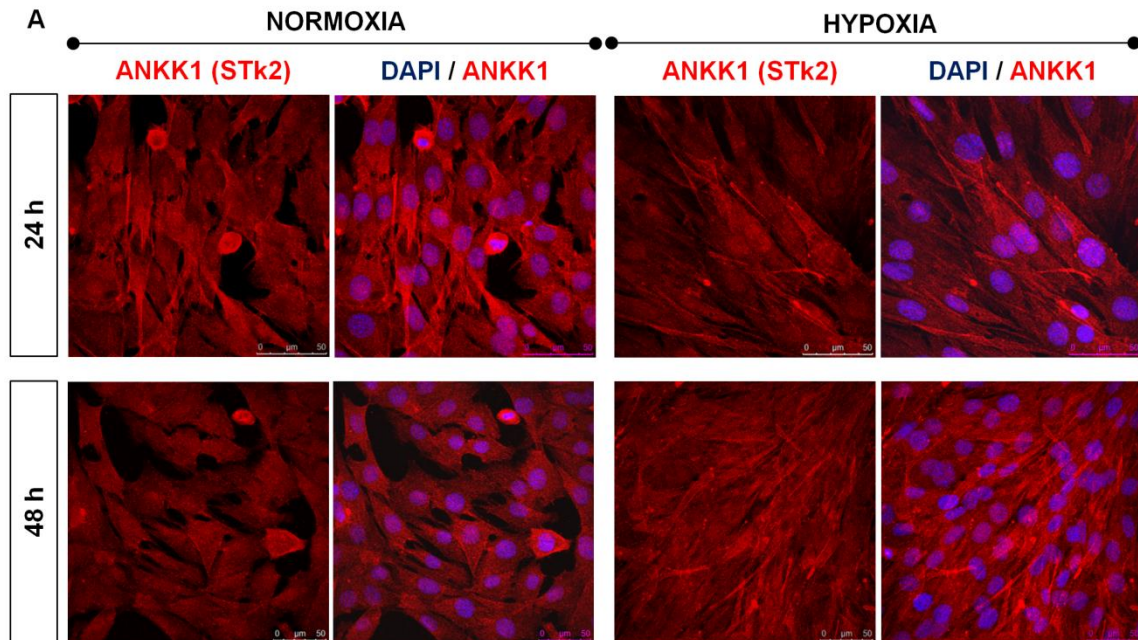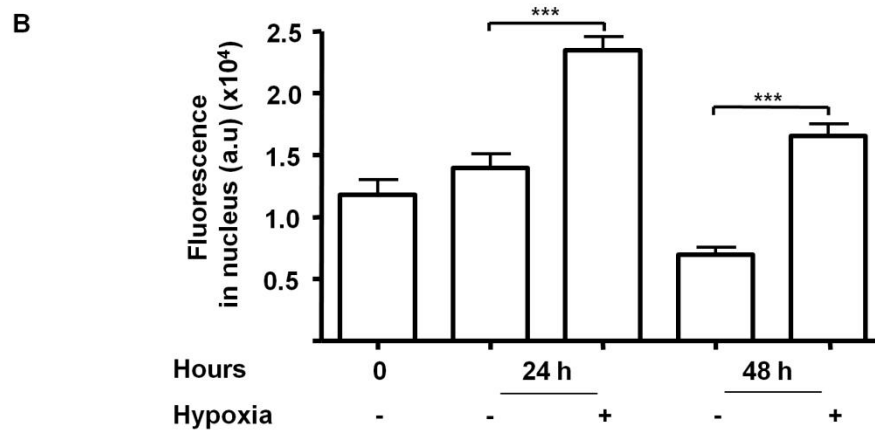

Supplement: S2 Fig — (A) ANKK1 (α-STk2) immunostaining of C2C12 proliferating myoblasts (24–48 h) in normoxia or hypoxia (2% O2). Images were taken from confocal optical sections that are representative for the group averages. (B) Quantification of the fluorescence intensity (a.u) of ANKK1 in nuclei (N = 3). p < 0.05: *; p < 0.01: **; p < 0.001: ***. a.u: arbitrary units. (PDF) [file pone.0197254.s002.pdf]
